# Systematic dysphagia screening and dietary modifications to reduce stroke-associated pneumonia rates in a stroke-unit

**DOI:** 10.1371/journal.pone.0192142

**Published:** 2018-02-01

**Authors:** Yvonne Teuschl, Michaela Trapl, Paulina Ratajczak, Karl Matz, Alexandra Dachenhausen, Michael Brainin

**Affiliations:** 1 Department for Clinical Neuroscience and Preventive Medicine, Danube University Krems, Krems, Austria; 2 Department of Neurology, University Hospital Tulln, Tulln, Austria; 3 Karl Landsteiner University of Health Sciences, Krems, Austria; Medizinische Universitat Innsbruck, AUSTRIA

## Abstract

**Background and purpose:**

While formal screening for dysphagia following acute stroke is strongly recommended, there is little evidence on how multi-consistency screening and dietary modifications affect the rate of stroke-associated pneumonia (SAP). This observational study reports which factors affect formal screening on a stroke-unit and how dietary recommendations relate to SAP.

**Method:**

Analyses from a database including 1394 patients admitted with acute stroke at our stroke-unit in Austria between 2012 and 2014. Dietary modifications were performed following the recommendations from the Gugging Swallowing Screen (GUSS). Patients evaluated with GUSS were compared to the unscreened patients.

**Results:**

Overall, 993 (71.2%) patients were screened with GUSS; of these 50 (5.0%) developed SAP. In the 401 unscreened patients, the SAP rate was similar: 22 (5.5%). Multivariable analysis showed that either mild to very mild strokes or very severe strokes were less likely to undergo formal screening. Older age, pre-existing disability, history of hypertension, atrial fibrillation, stroke severity, cardiological and neurological complications, nasogastric tubes, and intubation were significant markers for SAP. Out of 216 patients, 30 (13.9%) developed SAP in spite of receiving nil per mouth (NPO).

**Conclusion:**

The routine use of GUSS is less often applied in either mild strokes or very severe strokes. While most patients with high risk of SAP were identified by GUSS and assigned to NPO, dietary modifications could not prevent SAP in 1 of 7 cases. Other causes of SAP such as silent aspiration, bacteraemia or central breathing disturbances should be considered.

## Introduction

Pneumonia is a frequent complication after stroke and increases the risk for mortality and dependency [1–2]. Approximately 14% of patients suffer from pneumonia during the first week after stroke, but there is high variability in the reported numbers depending on the population, the study design and the diagnosis criteria [3]. Dysphagia is a major risk factor for stroke-associated pneumonia (SAP) observed in up to 78% of stroke patients, and has been associated with higher mortality, worse functional outcome and longer hospital stay [4–7].

There is some evidence from observational studies that formal screening for dysphagia following acute stroke can reduce the risk of pneumonia [8–13], and guidelines [14–15] strongly recommend screening of the swallowing abilities for all acute stroke patients as soon as patients are awake and alert.

We developed the Gugging Swallowing Screen (GUSS) in 2007 as a brief bed-side assessment screen for dysphagia and aspiration risk that can be used by speech and language therapists (SLT) as well as nurses [16]. In the meantime, the GUSS has been translated into 11 languages and has been further validated by use of fiberoptic endoscopic evaluation of swallowing (FEES) [17] and by an intervention study for use by trained nurses [13]. The GUSS allows a classification of the severity of dysphagia into 4 severity codes and provides nutritional recommendations accordingly.

In this study, we report the use of GUSS in clinical routine and how its dietary recommendations relate to dysphagia and the rate of SAP. We focus on three questions: 1) We analyse how many acute stroke patients were screened with GUSS at our stroke-unit and which were the factors associated with screening. 2) We analyse the frequency of dysphagia and the factors associated with its severity. 3) We report the frequency of SAP in relation to the use of GUSS and the prescribed diet.

## Materials and methods

This retrospective database analysis includes all patients (n = 1394) admitted with acute stroke from 2012 to 2014 to the acute stroke-unit at the University Clinic Tulln, Austria. All patients referred to the hospital under suspicion of stroke were directly admitted to the stroke-unit. The diagnosis of stroke was based on clinical presentation and brain imaging (computed tomography [CT] or magnetic resonance imaging [MRI]). A transient ischemic attack (TIA) was diagnosed if clinical symptoms of stroke lasted less than 24 hours and no lesion was detected on the CT or DW-MRI. All patients admitted to the stroke-unit were entered in the Austrian Stroke Unit Registry. The Austrian Stroke Unit Registry collects anonymized, stroke-relevant data on baseline characteristics, management and outcome of all stroke patients admitted to Austrian stroke-units (for more details see [18–19]). All aspects of data entry, data protection, administration and scientific analysis are regulated by law. A formal ethical approval from the local Austrian ethics committee was not needed. Data collection, ratings and data entry were performed by experienced stroke neurologists at the time of admission and discharge to the stroke-unit as well as via follow-up phone call three months thereafter. Stroke severity was assessed on admission and discharge from the stroke-unit using the National Institute of Health Stroke Scale (NIHSS). The modified Rankin Scale (mRS) was used to evaluate functional status before stroke, on admission and discharge from the stroke-unit as well as on follow-up three months later. Vascular risk factors were determined according to medical history, pre-stroke medication or were newly diagnosed during the stay at the stroke-unit. Stroke types were classified on the basis of neuroimaging findings and according to the International Classification of Diseases (ICD)-10 code into ischaemic stroke (I63 or I64) or haemorrhages (I60, I61 or I62). In line with previous evaluations in the acute stroke setting, standard diagnostic criteria were used for assessment of clinical complications [19].

Diagnosis criteria for pneumonia were based on the modified CDC criteria and the recommendations from the pneumonia in stroke consensus group for probable SAP [20]: clinical symptoms (e.g. cough, purulent sputum) in combination with clinical signs such as fever, rales, bronchial breath sounds or elevation of inflammatory markers in laboratory tests confirmed by at least one chest x-ray within 7 days after stroke. Pneumonia diagnosed later than 7 days after admission was defined as hospital associated pneumonia (HAP). Patients’ medical records which include all information (reports, examinations) of all medical wards during hospital stay were reviewed by a SLT. In cases with at least one chest x-ray, the reports from the radiologist, the internist and the final medical report from the neurologist were reviewed for a diagnosis of pneumonia during hospitalisation. When the reported diagnoses differed or did not correspond to the chest x-ray or to the reported symptoms and signs, the diagnosis of the internist was used. The occurrence of pneumonia after hospital discharge was not recorded during the 3-month follow-up.

Dysphagia screening was performed in accordance with stroke guidelines and the SOPs for Austrian Stroke Units: In brief, patients with tracheostomy and inflated cannula tubes or with reduced consciousness were restricted to receive nil per mouth (NPO). Patients with suspected impairment in cranial nerves involved in swallowing (V, VII, IX, X, XII), neurological diseases in the medical history, with neuropsychological deficits (e.g. apraxia, neglect) were tested by trained nurses or SLTs within 24h after admission with GUSS. All other patients were examined for swallowing disorders using a simple water swallowing test. Conspicuous patients are transferred to SLTs for further investigation and treatment.

Dietary modifications were performed according to the recommendations from the GUSS Score [16,21] and are described using the levels of the International Dysphagia Diet Standardisation Initiative (IDDSI) framework for texture-modified foods and thickened fluids [22]. As a consequence of the clinical experience gained over the years, an additional diet (NPO-med) was assigned under the supervision of the SLT to patients scoring 8 to 9 points on the GUSS: patients were not fed per mouth except for medications which were crushed and mixed with apple sauce. Data on GUSS (day of screening, GUSS score, recommended diet, profession of person performing the screening) were systematically recorded for all patients and entered in their medical records as part of the clinical therapeutic process either by the SLT or by the nurses performing the screening. As the GUSS is part of the clinical routine and data are analysed anonymously, no ethic approval is needed for the analysis of these data.

### Statistics

Univariate comparisons were made using the t-test for continuous normally distributed variables, the Mann Whitney U-test for continuous non-normally distributed variables and the Chi^2^ or the Fisher exact test for binary variables.

Patients evaluated with GUSS within 7 days were compared to the unscreened patients in a step-wise backward multivariable binary logistic regression analysis optimizing the AIC criteria including demographic factors, risk factors, stroke type, stroke severity (linear, quadratic), complications, treatment, delay of screening, day of screening, and stroke-unit mortality as explaining variables. The number of cases with pneumonia was low, making the multivariable models unstable. Therefore factors associated with pneumonia were only reported in a descriptive way by using univariate comparisons. The factors associated with dysphagia (<20 points on the GUSS) and with aspiration risk (<15 points) [16] were analysed in binary logistic regression models.

Because of the increasing risk of dysphagia starting at the age of 60 [23] the following categories were chosen for age: <60, 60–69, 70–79, 80–89, ≥90 years.

## Results

**Fig 1:**
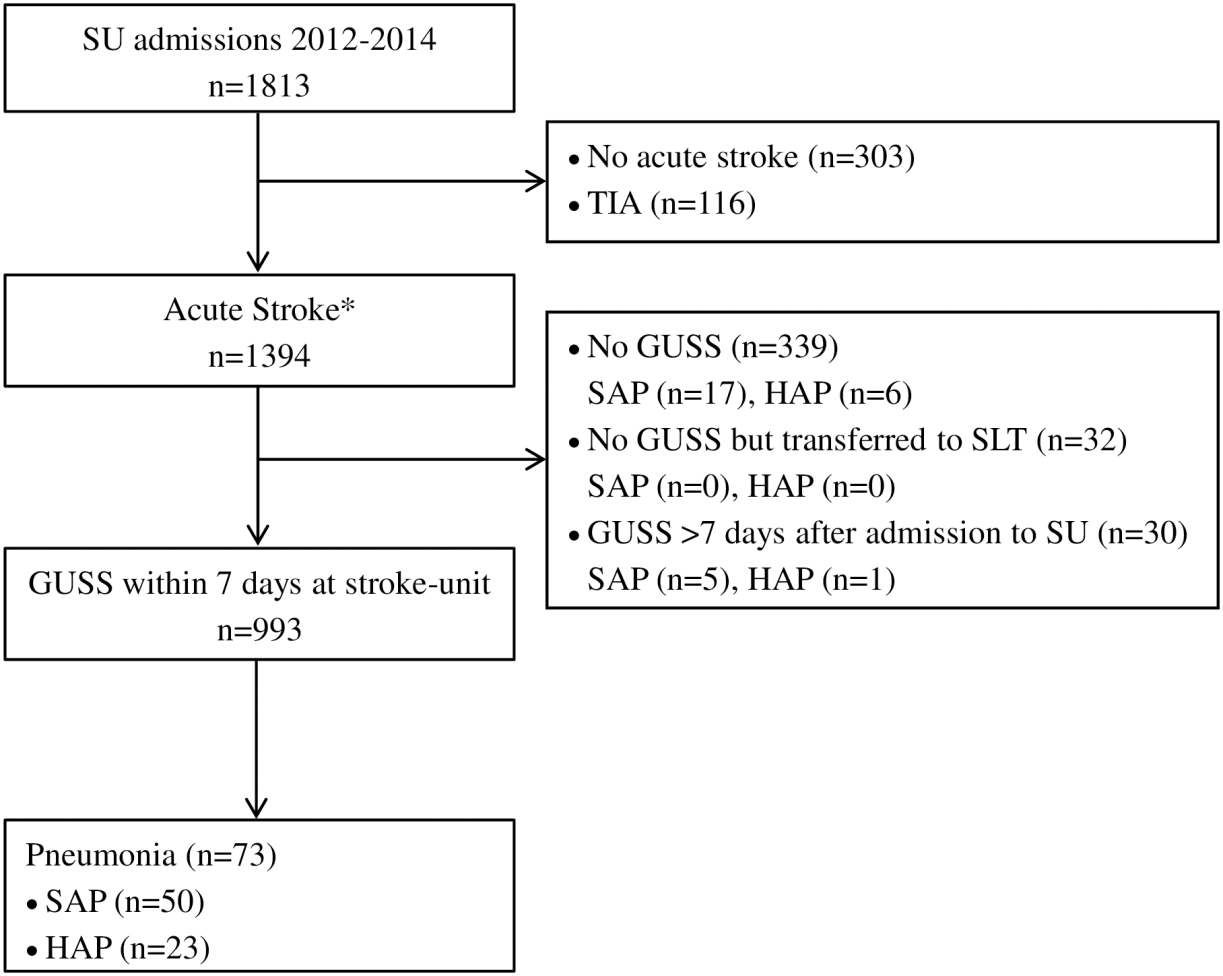
Patient flowchart. GUSS: Gugging Swallowing Screen, HAP: hospital-acquired pneumonia (>7 days post-stroke); SAP: stroke-associated pneumonia (≤7 days post-stroke); SLT: speech and language therapist, SU: stroke-unit. *According to the discharge diagnosis based on neuroimaging findings.

There were 1394 patients with acute stroke in the stroke registry between 2012 and 2014. Of these, 993 (72.2%) were tested with GUSS within the first 7 days. Of the remaining 401 patients 339 were not tested, 32 were transferred to SLTs for diagnostic reasons other than swallowing disorders (language disorders, facial paralysis) and 30 were tested more than 7 days after admission (Fig 1). Overall 899/1023 (88%) of all patients undergoing a GUSS were tested on the same or the next calendar day, and 939/1023 (92%) within the first three calendar days. The median stroke-unit stay was 2 days (IQR: 1 to 4).

### Factors associated with GUSS testing

Characteristics of the entire population as well as those tested with GUSS are shown in Table 1. The multivariable analysis showed that patients who were screened were older, had more often previous strokes, more often ischemic strokes, were more often admitted during working days, were more often treated with thrombolysis, suffered less from neurological complication and died less often on the stroke-unit (Table 2). Furthermore, the analysis showed a bimodal distribution of the stroke severity with either mild strokes or severe strokes being less likely to get screened (Table 2).

**Table 1:** Characteristics of patients undergoing (n = 993) and not undergoing (n = 401) the Gugging Swallowing Screen (GUSS) within 7 days.

Factors	No GUSS	GUSS	p-value^a^
**Demographic**			
Age	72 (59 to 82)	77 (67 to 84)	<0.001
*<60 years*	104 (25.9%)	144 (14.5%)	
*60–69 years*	74 (18.5%)	141 (14.2%)	
*70–79 years*	105 (26.2%)	299 (30.1%)	
*80–89 years*	99 (24.7%)	323 (32.5%)	
*≥90 years*	19 (4.7%)	86 (8.7%)	
Sex (female)	192 (47.9%)	479 (48.2%)	0.904
Pre-existing disability^b^	37 (9.2%)	175 (17.6%)	<0.001
**Risk factors**			
Hypertension	302 (75.5%)	776 (78.3%)	0.257
Diabetes	91 (22.8%)	233 (23.5%)	0.761
Previous stroke	46 (11.5%)	199 (20.1%)	<0.001
Myocardial infarction	30 (7.5%)	85 (8.6%)	0.509
Hypercholesterinaemia	153 (38.3%)	470 (47.4%)	0.002
Atrial Fibrillation	88 (22.0%)	289 (29.2%)	0.007
Other cardiac diseases	51 (12.8%)	161 (16.2%)	0.101
Peripheral arterial disease	19 (4.8%)	50 (5.0%)	0.818
Smoking	75 (18.8%)	161 (16.2%)	0.260
Alcohol	34 (8.5%)	81 (8.2%)	0.841
**Stroke related**			
mRS (admission)	3 (1 to 5)	4 (2 to 4)	<0.001
NIHSS (admission)	4 (1 to 16)	6 (3 to 12)	0.002
*0–6*	247 (61.6%)	527 (53.1%)	
*7–15*	50 (12.5%)	304 (30.6%	
*≥16*	104 (25.9%)	162 (16.3%)	
Barthel (admission)	70 (10 to 100)	55 (20 to 80)	0.001
Stroke type			<0.001
*Haemorrhagic*	77 (19.2%)	92 (9.3%)	
*Ischemic*	324 (80.8%)	900 (90.7%)	
**Complications SU**			
Neurological complications^c^	64 (16.0%)	67 (6.8%)	<0.001
Cardiac complications^d^	23 (5.8%)	30 (3.0%)	0.016
Other complications^e^	9 (2.3%)	15 (1.5%)	0.337
**Admission**			
Transferred from other hospital	111 (27.7%)	223 (22.5%)	0.039
Time stroke—admission SU (min)	165 (95 to 300)	145 (90 to 270)	0.188
SU admission on weekend	135 (33.7%)	264 (26.6%)	0.008
**Treatment**			
Thrombolysis i.v.	49 (12.3%)	219 (22.1%)	<0.001
Assisted ventilation	53 (13.3%)	105 (10.6%)	0.160
Intubation	25 (6.3%)	12 (1.2%)	<0.001
Nasogastric tube	71 (17.8%)	130 (13.1%)	0.027
Length of stay on SU (days)	1.0 (1.0 to 3.0)	3.5 (1.0 to 5.0)	<0.001
**Outcome**			
Pneumonia			0.761
*no pneumonia*	*372 (92*.*8%)*	*920 (92*.*6%)*	
*stroke associated pneumonia*	*22 (5*.*5%)*	*50 (5*.*0%)*	
*hospital associated pneumonia*	*7 (1*.*7%)*	*23 (2*.*3%)*	
Mortality SU	37 (9.2%)	15 (1.5%)	<0.001

Data are median (quartiles) or frequency (percentages)

i.v.: intravenous; LACS: lacunar syndrome; mRS: modified Rankin scale, NIHSS: National Institutes of Health Stroke Scale; SICH: symptomatic intracerebral haemorrhage; SU: stroke-unit;

^a:^ p-value for univariate group comparisons;

^b:^ mRS before stroke 3 to 5;

^c:^ Symptomatic intracerebral haemorrhage, cerebral oedema, epileptic seizure, progressive stroke or re-insult;

^d:^ Arrhythmia, cardiac decompensation or myocardial infarction;

^e:^ Sepsis, Urinary tract infection or extracerebrale haemorrhages

**Table 2:** Fal stepwise backward binary logistic regression model optimizing the AIC criterion for the factors associated with undergoing a Gugging Swallowing Screen within 7 days (R^2^ = 0.237, n = 1388).

Variable	Coefficient	SE	OR (95% CI)	p-value
Age <60^a^	-0.74	0.19	0.48 (0.33 to 0.69)	<0.001
Age 60–69	-0.44	0.20	0.64 (0.44 to 0.94)	0.024
Age 80–89^a^	0.10	0.18	1.10 (0.77 to 1.58)	0.599
Age ≥90^a^	0.64	0.33	1.90 (1.01 to 3.73)	0.053
MRS before stroke	0.32	0.20	1.37 (0.93 to 2.06)	0.118
Previous stroke	0.62	0.20	1.86 (1.27 to 2.77)	0.002
Stroke-unit mortality	-1.32	0.40	0.27 (0.12 to 0.58)	<0.001
Hypercholesterinaemia	0.21	0.13	1.23 (0.94 to 1.60)	0.125
Haemorrhagic stroke^b^	-0.52	0.21	0.59 (0.40 to 0.89)	0.011
Admission on weekend	-0.52	0.14	0.59 (0.45 to 0.78)	<0.001
Neurological complications ^c^	-0.82	0.23	0.44 (0.28 to 0.70)	<0.001
Cardiological complications^d^	-0.64	0.33	0.53 (0.28 to 1.02)	0.053
NIHSS	0.29	0.03	1.33 (1.25 to 1.43)	<0.001
NIHSS square	-0.01	0.00	0.99 (0.98 to 0.99)	<0.001
Thrombolysis i.v.	0.48	0.20	1.62 (1.11 to 2.40)	0.014

CI: confidence interval; i.v.: intravenous; mRS: modified Rankin scale, NIHSS: National Institutes of Health Stroke Scale; OR: odds ratio; SE: standard error; SU: stroke-unit

^a:^ reference group: age 70–79

^b:^ reference group: ischemic stroke

^c:^ Symptomatic intracerebral haemorrhage, cerebral oedema, epileptic seizure, progressive stroke or re-insult

^d:^ Arrhythmia, cardiac decompensation or myocardial infarction

For 20 of the 22 patients with SAP that were not tested with GUSS, possible reasons for not using the GUSS were identified: 2 patients died within 2 days, 6 were transferred to intensive care on the same or the next day, 14 were treated with nasogastric tubes, percutaneous endoscopic gastrostomy, intubated or had a tracheostomy, and 4 had an impaired level of consciousness on admission.

### Factors associated with dysphagia

**Fig 2:**
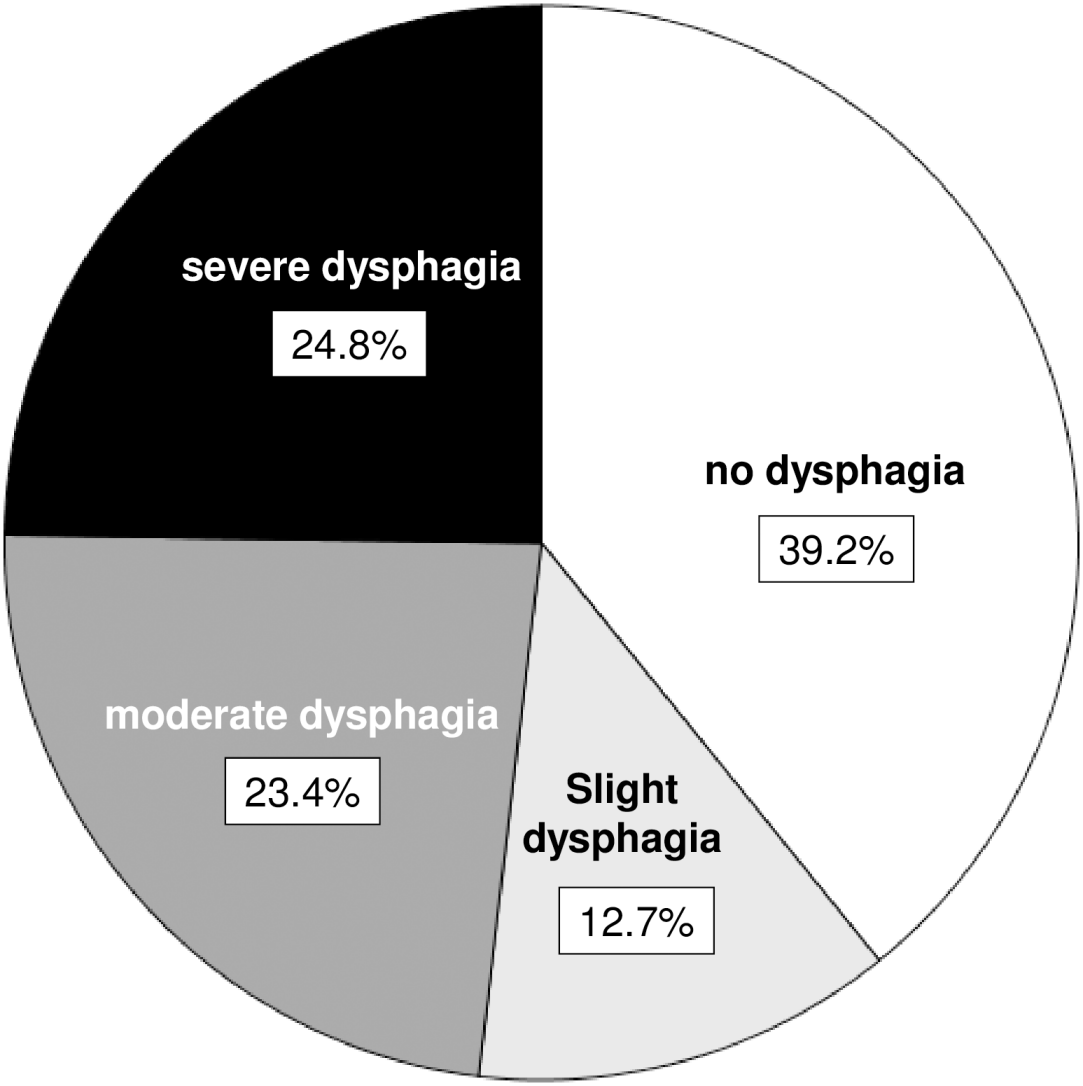
Severity of dysphagia according to the first Gugging Swallowing Screen (GUSS) score after admission at the stroke-unit: no (20 points), slight (15–19 points), moderate (10–14 points), severe (0–9 points) dysphagia.

The first GUSS was performed in 331/993 (33%) of the cases by nurses and in 662/1051 (67%) by a SLT in median on the day of admission to the stroke-unit (median: 0 days; IQR: 0 to 1). According to the first GUSS evaluation 389 (39.2%) patients had no sign of dysphagia (20 points), whereas 126 (12.7%) showed slight (15–19 points), 232 (23.4%) moderate dysphagia (10–14 points) and 246 (24.8%) severe dysphagia (0–9 points) (Fig 2). The presence of dysphagia (<20 points) was associated with older age, a worse functional status before stroke, diabetes, no stroke in medical history, haemorrhagic stroke, and more severe stroke (Table 3). The risk of aspiration (<15 points) was associated with older age, worse functional status before stroke, haemorrhagic stroke, and more severe stroke (Table 4).

**Table 3:** Final stepwise backward binary logistic regression model optimizing the AIC criterion for the factors associated with dysphagia (Gugging Swallowing Screen score <20 points; R^2^ = 0.41; n = 990).

Variable	Coefficientnt	SE	OR (95% CI)	p-value
Age <60^a^	-0.51	0.25	0.60 (0.37 to 0.98)	0.041
Age 60–69^a^	0.09	0.24	1.09 (0.69 to 1.73)	0.714
Age 80–89^a^	0.37	0.20	1.45 (0.97 to 2.17)	0.068
Age ≥90^a^	0.68	0.36	1.97 (0.98 to 4.07)	0.061
MRS before stroke	0.68	0.22	1.97 (1.28 to 3.05)	0.002
Diabetes mellitus	0.39	0.19	1.48 (1.02 to 2.16)	0.039
Previous stroke	-0.44	0.20	0.65 (0.43 to 0.96)	0.032
Haemorrhagic stroke^b^	0.83	0.29	2.30 (1.31 to 4.16)	0.004
NIHSS	0.33	0.05	1.39 (1.25 to 1.53)	<0.001
NIHSS square	-0.00	0.00	1.00 (0.99 to 1.00)	0.093

CI: confidence interval; mRS: modified Rankin scale, NIHSS: National Institutes of Health Stroke Scale; OR: odds ratio; SE: standard error

^a:^ reference group age 70–79;

^b:^ reference group ischemic stroke

**Table 4:** Final stepwise backward binary logistic regression model optimizing the AIC criterion for the factors associated with aspiration (Gugging Swallowing Screen score <15 points; R^2^ = 0.417; n = 990).

Variable	Coefficient	SE	OR (95% CI)	p-value
Age <60^a^	-0.89	0.27	0.41 (0.24 to 0.69)	0.001
Age 60–69^a^	-0.31	0.24	0.73 (0.45 to 1.17)	0.198
Age 80–89^a^	0.22	0.20	1.25 (0.85 to 1.83)	0.265
Age ≥90^a^	0.53	0.33	1.69 (0.90 to 3.23)	0.108
MRS before stroke	0.61	0.1911	1.84 (1.26 to 2.69)	0.002
Myocardial infarction	0.41	0.27	1.51 (0.88 to 2.58)	0.134
Smoking	0.38	0.22	1.46 (0.94 to 2.27)	0.089
Haemorrhagic stroke^b^	0.56	0.27	1.75 (1.04 to 2.98)	0.036
NIHSS	0.22	0.02	1.25 (1.21 to 1.29)	<0.001

CI: confidence interval; mRS: modified Rankin scale, NIHSS: National Institutes of Health Stroke Scale; OR: odds ratio; SE: standard error; TIA: transient ischemic attack

^a^ reference group age 70–79;

^b:^ reference group: ischemic stroke

### Factors associated with SAP

Overall 102 patients developed pneumonia (Fig 1). Of those 72 (4.8%) were classified as SAP: 22/401 (5.5%) in patients without GUSS and 50/993 (5.0%) in patients with GUSS. Due to the low numbers of SAPs, risk factors for SAP were only described by univariate comparisons (Table 5). Older age, pre-existing disability, history of hypertension, atrial fibrillation, stroke severity on admission, haemorrhagic strokes, cardiological and neurological complications, nasogastric tubes, and intubation were significant markers for the occurrence of SAP.

**Table 5:** Demographic factors, vascular risk factors, stroke related factors and treatment related factors in relation to incidence of stroke associated pneumonia (SAP).

Factors	No SAP (n = 1322)	SAP (n = 72)	p-value^a^
**Demographic**			
Age	75 (65 to 83)	80.5 (73 to 87)	<0.001
*<60 years*	244 (18.5%)	4 (5.6%)	
*60–69 years*	208 (15.7%)	7 (9.7%)	
*70–79 years*	381 (28.8%)	23 (31.9%)	
*80–89 years*	397 (30.0%)	25 (34.7%)	
*≥90 years*	92 (7.0%)	13 (18.1%)	
Sex (female)	641 (48.5%)	30 (41.7%)	0.259
Pre-existing disability^b^	195 (14.8%)	17 (23.6%)	0.041
**Risk factors**			
Hypertension	1013 (76.8%)	65 (90.3%)	0.008
Diabetes	310 (23.5%)	14 (19.4%)	0.428
Previous stroke	230 (17.4%)	15 (20.8%)	0.461
Myocardial infarction	105 (8.0%)	10 (13.9%)	0.075
Hypercholesterinaemia	593 (45.0%)	30 (41.7%)	0.584
Atrial Fibrillation	342 (25.9%)	35 (48.6%)	<0.001
Other cardiac diseases	195 (14.8%)	17 (23.6%)	0.042
Peripheral arterial disease	64 (4.9%)	5 (6.9%)	0.399
Smoker	227 (17.2%)	9 (12.5%)	0.300
Alcohol	107 (8.1%)	8 (11.1%)	0.368
**Stroke related**			
mRS (admission)	4.0 (2.0 to 4.0)	4.0 (4.0 to 5.0)	<0.001
NIHSS (admission)	5.0 (2.0 to 12.0)	13.5 (6.5 to 20.0)	<0.001
*(0–6)*	756 (57.2%)	18 (25.0%)	
*(7–15)*	333 (25.2%)	21 (29.2%)	
*(≥16)*	233 (17.6%)	33 (45.8%)	
Barthel (admission)	60.0 (20.0 to 90.0)	20.0 (0.0 to 60.0)	<0.001
Stroke type			0.002
*Haemorrhagic*	152 (11.5%)	17 (23.6%)	
*Ischemic*	1169 (88.5%)	55 (76.4%)	
**Complications SU**			
Neurological complications^c^	117 (8.9%)	14 (19.4%)	0.003
Cardiological complications^d^	42 (3.2%)	11 (15.3%)	<0.001
Other complications^e^	22 (1.7%)	2 (2.8%)	0.356
**Admission**			
Transferred from other hospital	323 (24.4%)	11 (15.3%)	0.076
time stroke—admission SU (min)	150.0 (90.0 to 285.0)	105.0 (75.0 to 180.0)	0.008
SU admission on weekend	378(28.6%)	21 (29.2%)	0.916
**Treatment**			
Thrombolysis i.v.	257 (19.5%)	11 (15.3%)	0.377
Assisted ventilation	146 (11.1%)	12 (16.7%)	0.146
Intubation	31 (2.4%)	6 (8.3%)	0.010
Nasogastric tube	171 (13.0%)	30 (41.7%)	<0.001
Length of stay on SU (days)	2.0 (1.0 to 4.0)	4.0 (2.0 to 6.0)	<0.001
**GUSS** ^f^			
time stroke—GUSS (days)	1 (0 to 1)	1 (0 to 1)	0.222
time SU admission—GUSS (days)	0 (0 to 1)	0 (0 to 1)	0.214
First GUSS on weekend	192 (20.4%)	8 (16.0%)	0.454
First GUSS by nurse	320 (33.9%)	11 (22.0%)	0.081
GUSS (points)	17 (12 to 20)	8 (2 to 13)	<0.001
**Outcome**			
Death SU	46 (3.5%)	6 (8.3%)	0.048
Death 3 months^g^	130 (14.2%)	25 (55.6%)	<0.001

Data are median (quartiles) or frequency (percentages)

i.v.: intravenous; mRS: modified Rankin scale, NIHSS: National Institutes of Health Stroke Scale; SICH: symptomatic intracerebral haemorrhage; SU: stroke-unit

^a:^ p-value for univariate group comparisons;

^b:^ mRS before stroke 3 to 5;

^c^: Symptomatic intracerebral haemorrhage, cerebral oedema, epileptic seizure, progressive stroke or re-insult;

^d:^ Arrhythmia, cardiac decompensation or myocardial infarction;

^e:^ Sepsis, urinary tract infection or extracerebrale haemorrhages;

^f:^ Subsample of patients screened with GUSS within 7 days (n = 993);

^g:^ Subsample of patients with follow-up who were alive when discharged from the stroke-unit (n = 918)

### GUSS, dietary modifications and SAP

SAP was highest in patients with severe dysphagia according to GUSS scores: 3/389 (0.8%) patients with normal swallowing functions, 3/126 (2.4%) with slight, 12/232 (5.2%) with moderate, and 32/246 (13.0%) with severe dysphagia developed SAP.

The diet that was prescribed to the patients was in accordance with the recommendations of the GUSS. Only 6 of the 993 patients with GUSS received a diet that was less strict than the recommended diet (Table 6). None of these patients developed SAP. Another 33 patients with severe dysphagia were assigned to NPO-med, i.e. they were assigned to NPO but received their medication orally, crushed and mixed with apple sauce. Two of these patients developed SAP. Overall, 30 of the 50 (60%) patients who developed SAP were assigned to receive NPO (Table 6). In 41 of the 50 (82%) patients with SAP the GUSS was performed on the same or the next calendar day.

**Table 6:** Diet that was administered according to the severity of dysphagia, measured with the Gugging Swallow Screen (GUSS). The number of stroke associated pneumonia (n = 50) are presented in parentheses; grey cells represent diet recommendations according to GUSS, cells above the grey cells indicate a diet less strict, cells below a diet stricter than recommended by GUSS; spotted cell indicate 8–9 points on the GUSS and administered to NPO-med diet.

	Severity of dysphagia^a^			
Diet	No	Slight	Moderate	Severe
Normal	377 (3)	5	0	0
Minced & moist^b^	12	93 (3)	0	0
Pureed^c^	0	27	218 (9)	1
NPO-med^d^	0	0	11 (3)	33 (2)
NPO	0	1	3	212 (30)

NPO: nil per mouth.

^a^According to the first GUSS after admission at the stroke-unit: no (20 points), slight (15–19 points), moderate (10–14 points), severe (0–9 points) dysphagia.

^b:^ Dysphagia diet (minced & moist or soft & bite-sized, level 5 or 6 according to IDDSI Framework); liquids thickened, level 1 or 2 according to IDDSI Framework)

^c:^ Pureed textures (level 3 or 4 according to IDDSI Framework); all liquids must be thickened (level 2 or 3 according to IDDSI Framework)

^d:^ NPO except crushed medication mixed with apple sauce

### Mortality

Overall, 52 (3.7%) patients died on the stroke-unit. Mortality was higher among patients with SAP (8.3%) compared to those without (3.5%) (Table 5). Of those patients alive at discharge from the stroke-unit with a follow-up (n = 918) another 155 (16.9%) died during the next 3 months; 25 (55.6%) of those with SAP compared to 130 (14.2%) without SAP (Table 5).

## Discussion

In this cohort of patients admitted with acute stroke or TIA to a stroke-unit, 72% were tested with the GUSS. The GUSS was less often applied in mild strokes as well as in very severe strokes. The GUSS identified patients with the highest risk of SAP and they were assigned to NPO. However, dietary modifications could not prevent pneumonia in all stroke cases—especially not in patients who had already developed severe dysphagia.

### Use of formal dysphagia screening

In clinical centres following recommendations for formal dysphagia screening the percentages of patients screened after acute stroke varied between 69% and 88% which is comparable to the 72% of patients tested with GUSS in our study [8,11,24–26]. Unfortunately, we did not systematically document the reasons for not using a GUSS and thus the number of patients ineligible for testing. In our study, dysphagia screening was not performed when patients were strongly affected, probably making early testing impossible, or in very mild cases where no pneumonia was expected. This is comparable to the results of a German stroke registry where two groups of patients were identified who did not undergo dysphagia screening: younger and less affected patients, as well as patients with highly impaired consciousness [25]. Similarly two other observational studies showed that patients with more severe stroke were more likely to be screened [9,26]. Patients with mild strokes are at lower risk of dysphagia and pneumonia. Nevertheless, 2.3% of our patients with mild strokes (NIHSS score ≤6) developed pneumonia, 130 of 527 (25%) were at risk of aspiration (GUSS score <15), and 203/527 (39%) had dysphagia (GUSS score <20; data not presented). Similarly, 33% of mild strokes failed dysphagia screening in the Ontario stroke registry [26]. These percentages might be overestimated because patients with mild stroke and clinical signs of swallowing disorders may be more likely to be screened. However, given that dysphagia is not included in the NIHSS and because of possible pre-existing swallowing disorders in elderly patients, mild strokes should not be omitted from dysphagia screening.

The GUSS has been validated to be used by SLT as well as by nurses [16]. Indeed our study showed that the first GUSS was performed in 35% of cases by nurses. A recent study conducted on another Austrian Stroke-Unit showed that the systematic training of nurses to perform the GUSS decreased the rate of pneumonia from 11.6% to 3.8% [13]. This underlines the importance of an interdisciplinary bedside screen such as GUSS which can be used on weekends, holidays and outside the regular working hours in the absence of a SLT.

### Incidence of dysphagia

Of those patients undergoing a screening 61% were diagnosed with dysphagia according to the first GUSS. The rate is comparable to an incidence rate of 30% to 55% found in a review when only clinical testing is used after acute stroke [4]. The rate of dysphagia found in our study might over- or underestimate the incidence of dysphagia as mild and severe stroke were less likely to be screened. Furthermore, as in other studies, pre-existing swallowing impairment was not assessed. Apart from pre-stroke conditions, anatomical, physiological, psychological, and functional changes as part of “normal aging” contribute to alterations in swallowing in persons older than 60 years [23]. However, swallowing disorders in the apparently healthy elderly population often occur without clinical complaints [27], and it may thus be difficult to evaluate them retrospectively after the stroke event.

### Prevalence of SAP

As previously found in other studies, the rate of SAP was higher for patients with dysphagia compared to patients with normal swallowing function [4,6,25,26], and was highest for those with severe dysphagia.

Early interventions such as dietary adaptations, intensive swallowing therapy and oral hygiene can reduce the incidence of chest infections in stroke patients with dysphagia [28,29]. In our study, 5.2% of patients developed SAP. Compared to overall rates of post-stroke pneumonia of 10% and 14% in two recent meta-analyses [3,30] this rate is relatively low and suggests that the dietary modifications recommended by the GUSS may be successful in preventing SAP. However, there is high variability in the reported numbers of pneumonia depending on the population, the study design and the diagnosis criteria. In a multi-centre study formal dysphagia screening was found to lower pneumonia rate as low as 2.4% compared to 5.4% in sites with no formal screening [10]. Similarly, the implementation of a dysphagia screen increased the percentage of patients being screened from 39% to 74% and decreased HAP from 6.5% to 2.8% in a prospective single-centre study [9]. In a Japanese single-center study the implementation of a multidisciplinary swallowing team decreased the rate of SAP from 16% to 7% [12]. Recent analyses of stroke registers of centers following national guidelines recommending dysphagia screening in acute stroke may be more comparable to our study and reported pneumonia rates between 4% and 10% [8,11,24–26].

As in previous studies, more severe strokes, older age and impairment before stroke were associated with dysphagia as well as with SAP [6,26]. Indeed, these three variables are key elements of SAP prediction scores such as the A2DS2, the AIS-APS or the integer-based pneumonia risk score (ISAN) [31]. We did however not find an association between sex and pneumonia.

### Dietary modification and SAP

While the GUSS was validated as bed-side screening instrument to identify acute stroke patients at risk of aspiration and dysphagia, the nutritional recommendations were not tested for their ability to prevent SAP [16].

The rate of SAP was not lower in the group of patients screened with GUSS. A similar result was found by Titworth et al 2013 who found that after the implementation of a dysphagia screen the rate of pneumonia decreased but was not different between patients that were screened and those that were not [9]. This may be explained by screening being more likely in more severe strokes and thus masking the positive effects of interventions. In the majority of patients that were not screened with GUSS but developed SAP we identified reasons which might have prevented early testing or which show that patients have been assigned to NPO anyway—independent of a GUSS. It is thus probable that the GUSS and its diet recommendations would not have prevented SAP in those patients.

As recommended by the GUSS, patients with severe dysphagia were assigned to NPO—nevertheless 60% of all SAPs occurred despite of NPO. A retrospective database analysis of Australian hospitals showed a similar result: NPO and nasogastric tubes were found to be predictors for respiratory infections. Respiratory infection developed in 37% of patients with nasogastric tube compared to 5% without [32]. Similarly, in our study 15% of patients with nasogastric tube developed SAP compared to 4% without. It has been suggested that nasogastric tubes might not only decrease the risk of aspiration by lowering the risk of aspiration during eating but may as well increase the risk of respiratory infections due to a higher bacterial load of the saliva [33]. However, studies were not randomized, and the causal relationship remains unclear. Indeed 96% of patients with nasogastric tubes in the study of Brogan et al. [32] and 94% in our study were dysphagic. Furthermore, additional factors that may influence the development of respiratory infections in patients with nasogastric tubes such as oral hygiene, immobility or additional treatment with antibiotics were not documented systematically in the current study. Another important factor which was not recorded was the timing of nasogastric tube insertion. It has been suggested that the risk for respiratory infections is highest in the first 3–4 days post-stroke and that other methods (e.g. intravenous) might be used during this time before starting with enteral feeding [33].

Apart from assigning patients with severe dysphagia to NPO, the multiconsistency screening GUSS is the only screening instrument that recommends special diets for patients with slight or moderate dysphagia. Compared to a water test which would have assigned these patients to NPO because of problems with swallowing liquids, patients with moderate dysphagia (218/993; 22%) were instead assigned to a special diet thereby increasing the quality of life for those patients. Only 9/218 (4%) developed SAP in this group. Furthermore, the data suggest that it may be safe to give medication orally, crushed and mixed with apple sauce to patients with severe dysphagia (8–9 points on the GUSS), as only 2/33 (6%) developed SAP compared to 30/212 (14%) of NPO patients. This allows parenteral nutrition in the first 2–3 days after the event in patients not being at risk for malnutrition—avoiding thereby nasogastric tubes [34].

### Limitations

The main limitation of this study is its single centre retrospective observational design. The stroke-unit registry was established prospectively and data were collected with standardized methods. The occurrence of pneumonia was however analysed retrospectively using diagnoses from medical records and was limited to the time of hospitalization.

Despite that, the majority of screenings were performed on the day of admission, the exact time of dysphagia testing was not recorded. Delay in dysphagia screening during the first 8 hours has however been associated with SAP risk [8]. Further limits were missing variables such as ineligibility for GUSS, speech language therapy, other stroke management factors such as oral hygiene or treatment with antibiotics.

Swallowing functions vary during the first week post-stroke. In practice, SLTs are therefore screening patients several times, include more comprehensive dysphagia assessments such as FEES and adapt constantly the diet recommendations accordingly. Analysing only the first GUSS that was performed after admission and the according diet prescription may therefore not reflect the whole picture.

### Conclusion

In clinical routine the GUSS is applied in 72% of patients and, if eligible, the majority of those who later developed pneumonia were screened. Despite of an identification of patients at risk by the GUSS and the use of dietary modifications, 5.2% of patients still developed SAP. Additional to diet other management factors such as timing of nasogastric tubes, oral hygiene or antibiotics may help to further decrease the rate of SAP.

## Supporting information

S1 File Dataset. (CSV)
